# Frontal Eye Field Involvement in Color and Motion Feature-Based Attention: Single-Pulse Transcranial Magnetic Stimulation

**DOI:** 10.3389/fnhum.2018.00390

**Published:** 2018-10-01

**Authors:** Xi Chen, Jing-Na Jin, Fang Xiang, Zhi-Peng Liu, Tao Yin

**Affiliations:** ^1^Institute of Biomedical Engineering, Chinese Academy of Medical Sciences & Peking Union Medical College, Tianjin, China; ^2^Neuroscience Center, Chinese Academy of Medical Sciences, Beijing, China

**Keywords:** transcranial magnetic stimulation, frontal eye field, feature-based attention, color, motion

## Abstract

An object can have multiple attributes, and visual feature-based attention (FBA) is the process of focusing on a specific one of them. During visual FBA, the frontal eye field (FEF) is considered to be an important brain area related to the choice of attribute. However, the study of the FEF in FBA remains inadequate. We applied single-pulse transcranial magnetic stimulation (TMS) to the right FEF (rFEF), and designed two independent experimental FBA tasks that each involved two attributes (color and motion), to explore the action time of FEF and the spatial transmission of the FEF signal, respectively. The results of the first experiment showed that when TMS was applied to the rFEF at 100 ms after the target image stimulus began, the subjects’ response time increased significantly compared with the response time in the control trials (in which TMS was applied to the vertex). This indicated that inhibiting the rFEF influenced the progress of visual FBA. The results confirm that the FEF is involved in the early stage of visual attention (at ~100 ms). In the second experiment, TMS was applied at 100 ms after the target image stimulus began. We analyzed the electroencephalogram (EEG) signal after TMS, and found that the electrode signal amplitudes for FC4 (which corresponded to the rFEF) were significantly correlated with the electrode signal amplitudes in the posterior regions. In addition, the amplitude rise of the posterior electrode signal lagged ~50 ms behind that of the FC4. Furthermore, for color and motion, different areas in the posterior brain region were involved in signal transmission. In this study, the application of single-pulse TMS was shown to provide a direct and effective method for research on the FEF, and the combination of TMS and EEG recordings allows a high degree of time resolution, which can provide powerful evidence for research on neural signal transmission.

## Introduction

The frontal eye field (FEF) is an area located near the junction between the anterior central sulcus and the posterior superior frontal sulcus (Paus, 1996), corresponding to Brodmann area 8. The FEF is an important brain area that has been reported to control eye movement (Bosch et al., 2013). Also, recent research has shown that the FEF is involved in visual attention, together with the posterior parietal cortex (PPC) and prefrontal ventral cortex (PFv; Lane et al., 2011). Muggleton et al. (2011) conducted a comparative experiment on the FEF and PPC, and they found that the FEF was focused on the process of visual attention and accumulation of visual information, while PPC participated in the transformation of visual information to behavior. A similar result was obtained by Akaishi et al. (2013). The studies on the FEF can be divided into two main categories: exploring the action time of the FEF and exploring the function of the FEF with regard to the top-down signal from the frontal cortex.

Previous experiments (Kammer, 2007; Romei et al., 2007), have shown that the FEF plays a major role prior to initial processing in the primary visual cortex at the early stages of visual attention. More specifically, neurons in the FEF of monkeys have been shown to discriminate the target and interference stimuli since about 100 ms from visual stimulus began (Bichot and Schall, 1999). O’Shea et al. (2004) applied paired-pulse transcranial magnetic stimulation (TMS) to subjects at different time periods during visual attention, and they found that paired-pulse TMS at 40–80 ms after the beginning of the target stimulus suppressed the subjects’ responses.

During visual attention, the transmission of visual information is thought to be controlled by a set of top-down signals from the frontal cortex, and the FEF is one of the important brain regions involved in the transmission. Recently, studies have demonstrated the transmission of the FEF signal to the posterior region of the brain, with the sequence of neural activity first involving the anterior and then the posterior brain regions (O’Shea et al., 2004; Brass et al., 2005). Further research has also shown that the correlation between the activities in the anterior and posterior brain regions demonstrates that the signal passes between them (Sakai and Passingham, 2003, 2006). In 2009, Morishima et al. proposed a single-pulse TMS method that involved the superposition of a nerve signal at the stimulation site and subsequently analyzing the transfer of this superimposed signal (Morishima et al., 2009). This allowed them to effectively prove that, when facial and motion information was being assessed, the signal transmission mechanism involved a top-down signal (Morishima et al., 2009). In 2014, Heinen et al. further proved this point using TMS combined with functional magnetic resonance imaging (fMRI; Heinen et al., 2014).

Both groups investigated the effect of the FEF during visual attention related to different tasks. However, regarding feature-based attention (FBA), attention is focused on a specific attribute of a single target, such as color, shape, or size (Treisman and Gelade, 1980; Tsujimoto and Tayama, 2004; Cavina-Pratesi et al., 2010), ignoring the other attributes. At present, there is not enough evidence on the function and action time of the FEF during FBA.

In visual FBA, the processing of different attributes is thought to correspond to different brain regions. In recent years, a large number of imaging and electrophysiological studies have provided evidence on the visual pathway involved in FBA. Studies have shown that the brain area corresponding to color information processing is located at V4/V8 in the occipital cortex (Pasupathy and Connor, 1999; Bichot et al., 2005; Zhou and Desimone, 2011). In contrast, the processing of motion information corresponds to the V5/MT region (Schoenfeld et al., 2007; Buracas and Albright, 2009; Alexander et al., 2018). However, whether this process is related to the FEF remains unknown.

Therefore, we designed two independent FBA experiments to explore the action time of the FEF and the spatial transmission of the FEF signal, respectively. The two experiments both involved applying single-pulse TMS to the right FEF (rFEF). The experiments only investigated the rFEF because research shows that rFEF has a hemispherical advantage over the left side (Marshall et al., 2015), i.e., the contribution of the right side to the attention process is greater than that of the left side.

In the first experiment, we set the stimulus interval between TMS and the beginning of the visual target stimulus to 0, 50, 100, 150 and 200 ms. Single-pulse TMS above the stimulation threshold was applied to the rFEF. Based on the experimental design used by Pourtois et al. (2001) for studying the PPC action time, the role of the FEF in FBA was explored by analyzing the response time of subjects as TMS was applied at different time points.

In the second experiment, we applied single-pulse TMS below the stimulation threshold at the time point associated with the maximum effect of the FEF (as shown in the first experiment) in order to facilitate an analysis of FEF signal transmission. The single-pulse TMS resulted in the superposition of a neural signal at the stimulated brain region (i.e., the FEF) that did not change the FEF’s function, and then the FEF’s function was studied by analyzing the subsequent spread of the superimposed signal.

## Materials and Methods

### Subjects

Nine normal subjects (mean age: 26.3 ± 3.2 years) participated in the first experiment to explore the action time of the FEF. We excluded one subject’s data as he could not concentrate on the visual FBA task for a sufficient length of time. In addition, 14 subjects (mean age: 26.1 ± 2.8 years) participated in the second experiment, which involved TMS combined with electroencephalogram (EEG) recording. The purpose of the second experiment was to explore the spatial transmission of the FEF signal. This study was carried out in accordance with the recommendations of TMS safety instructions, the ethics committee of the Institute of Biomedical Engineering, Chinese Academy of Medical Sciences & Peking Union Medical College. The protocol was approved by the ethics committee of the Institute of Biomedical Engineering, Chinese Academy of Medical Sciences. All subjects gave written informed consent in accordance with the Declaration of Helsinki.

### Task

**Figure 1:**
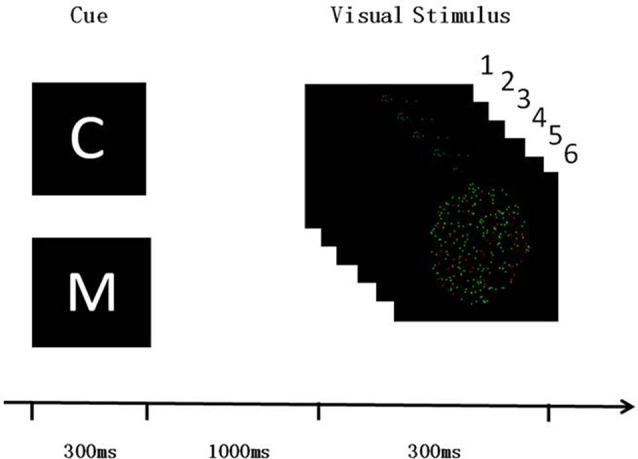
Visual feature-based attention (FBA) task paradigm. Both of the experiment 1 and the experiment 2 used the same design paradigm. Subjects needed to discriminate either the color or the direction of moving for most of dots in the visual stimulus dynamic pictures. C and M cues indicated color and motion discrimination, respectively. The stimulus onset asynchrony between the cue and visual stimulus was 1,000 ms, and the screen kept black during this time.

The paradigms of the two experiments were the same. Subjects were presented with an FBA task that involved assessing a specific visual attribute depending on a specific cue letter (C for color or M for motion; Figure 1). The refresh rate of the liquid-crystal display (LCD) monitor was 60 Hz. The room was kept dark and quiet during the experiment, to ensure that the subjects could focus on the FBA task. The task was written using E-prime software (E-prime2.0, Psychology Software Tools Inc., Sharpsburg, PA, USA). The subjects were required to assess the visual target information according to the cue letter. The visual target information was composed of six pictures, each of which were presented for 50 ms, making up a dynamic image stimulation. Two-hundred dots that were evenly distributed in the 6° of visual field located in the middle of the picture which was black background. The dots were randomly colored either red or green, which were set the same brightness and contrast, and they moved at 12°/s to the left or right. 20% of the dots were used as interference, i.e., they were different from the color or direction of motion of the other dots. The subjects were required to assess either the color or the direction of motion of most of the dots. That is, if the cue was C, subjects were required to assess the color of most of the dots, pushing button 1 for red and button 2 for green. If the cue was M, the subjects were required to assess the direction of motion, pushing button 1 when most dots move to the left and button 2 when they move to the right.

### TMS

For each subject, we first determined the TMS threshold intensity. Rest motor threshold (RMT) and active motor threshold (AMT) were measured individually for the first and second experiments. We defined RMT and AMT as the minimum TMS intensity that led to at least five electromyography signals being recorded in 10 successive TMS trials. For assessing the RMT, the subjects were seated on a chair with their right hand in a resting position, and for assessing the AMT, their right index finger was extended and lifted up. We delivered single-pulse TMS to the scalp position corresponding to the left primary motor cortex (the position was adjusted by moving the coil center in intervals of 0.5 cm) using an 8-shaped flat coil (Magstim, Whiteland, UK). A 70-mm coil was used for TMS, placing it tangentially over the scalp at 45° from the middle line.

### Site Localization

A structural MRI scan (T1) was obtained in advance for all subjects at Tianjin Medical University General Hospital. Stimulation sites for TMS were localized using the Brainsight system (Brainsight, Magstim, UK). This was used to match each subject to their MRI scan, on which the rFEF and vertex were marked before the experiments. The stimulation site in both the first and second experiments was set as the rFEF coordinates of Montreal Neurological Institute (MNI; 28 ± 4, −5 ± 5, 49 ± 4), corresponding to the coordinates of the rFEF reported in previous study (Paus, 1996).

### Experiment 1

In the first experiment, single-pulse TMS (110% RMT) was applied, and the stimulation times were set to 0, 50, 100, 150 and 200 ms from the beginning of the first visual target stimulus (see Figure 1, each time point corresponds to the beginning of one of the pictures). TMS was applied to the rFEF and vertex in the experimental and control trials, respectively. The first experiment had 10 blocks, each containing 80 trials, leading to a total of 800 trials. Subjects rested for 5 min in the interval between each block. Regarding the 10 blocks, there was a 5 × 2 design (time points 0/50/100/150/200 ms and stimulation site FEF/vertex). The time point and stimulation site used for each block were both set randomly, and C/M were also set randomly in each block.

### Experiment 2

In the second experiment, single-pulse TMS (70% AMT) was applied, and the stimulation time was set to the time corresponding to the maximum FEF effect obtained in the first experiment. The experiment had 10 blocks, each containing 64 trials, leading to a total of 640 trials. The experiment had a 2 × 2 design (TMS/no TMS and C/M). The TMS and no TMS trials occurred randomly.

### EEG Recording and Data Analysis

Subject wore an EEG cap (Neuroscan, Compumedics, USA) and performed the color or motion FBA task while EEG recordings were obtained from 60 scalp electrodes. Two additional electrodes were used to record the electrooculographic (EOG) which used in removing the artifacts of eye movement and blinking for further EEG analysis. EEG signals were referenced to FCz and the ground electrode was at Afz, signals filtered at a frequency of 200 Hz DC and sampled at 20 kHz. To reduce the impact of artifacts resulting from the clicking sound of the TMS pulse, the subjects wore earplugs. After discarding the raw data from trials involving incorrect color/motion assessments, we used EEGLAB toolbox version 13.0 (EEGlab, SCCM, San Antonio, TX, USA) combined with Matlab version 10.0 (Matlab, Mathworks, Natick, MA, USA) to process the EEG data, resample the data at 1,000 Hz, and an interpolation method (Rogasch et al., 2014) was applied to remove the TMS pulse induced artifacts, during the time interval of 40 ms before and after TMS (−20 ms to 20 ms). After interpolation algorithm, the EEG signals was filtered at 1–70 Hz and the reference was then changed to the average of the 60 electrodes, and the baseline was set to 20–40 ms before the TMS pulse was applied. Independent component analysis (ICA) was run two times to remove other artifacts, such as interpolation induced artifacts, TMS induced electromyogram (EMG) and blinking artifacts (Bai et al., 2016) during experiments. In the first ICA run, we identify the artifacts through components signal analysis combined with topographies, and further validate signals by statistical test after the second ICA run to remove the residual artifacts. After preprocessing the EEG data, the TMS-event-related potential (ERP) and no TMS-ERP signals were obtained separately for further analysis.

### Eye Movement Recording

In the second experiment, we used two additional electrodes to record horizontal and vertical eye movement signals. A Tobii (X1) eye tracker (Tobii, Sweden) was also used to record eye movement during the visual FBA task. The eye movement instrument was combined with the visual stimulation software (Eprime) to ensure synchronous measurements. The eye movement information was mainly recorded during visual target stimulation (~300 ms) in both experiments, which included the binocular position coordinates and pupil diameter. The data were then analyzed to examine the subjects’ attention.

### Analysis of Variance (ANOVA)

Analysis of variance (ANOVA) is a statistical procedure for summarizing a classical linear model which originally was developed by Fisher (1925). The classical idea of the ANOVA was to find out if there exists an influence of one or more factor variables (one factor in our experiment) over a normally distributed random variable. In the first experiment, we applied one factor variance analysis to explore the influence of two groups which included feature attributes (color and motion) and TMS stimulus sites (FEF and vertex) on the response time of subjects, individually, and the influence between the two groups.

### Pearson Correlation Coefficient Analysis

After data preprocessing, the difference in terms of TMS-ERP minus no TMS-ERP signal was used to calculate the Pearson correlations between the electrode signal of FC4 (near the rFEF) and the electrodes located in the parietal lobe and posterior region. The Pearson correlation coefficient was used to determine the degree of correlation between the electrode signal amplitudes. Coefficients of −1 and 1 represent perfect correlation, and the closer each coefficient was near to −1/1, the correlation was stronger.

### Current Source Density Analysis

Standardized low-resolution brain electromagnetic tomography (sLORETA) is a source location method based on a mathematical model (Fuchs et al., 2002; Pascual-Marqui, 2002; Wagner et al., 2004; Jurcak et al., 2007). By assessing the EEG signal in order to locate the activity source in the cerebral cortex, neural activity and signal transmission can be analyzed spatially. sLORETA software is based on a probabilistic MNI brain volume scanned at a resolution of 5 mm. In our study, data were calculated point by point by sLORETA at 20–50 ms after TMS, and the data were then normalized after log conversion. The images in each section were then superimposed, one by one and averaged. To assess the difference between the color and motion attributes, sLORETA results for color and motion were analyzed using paired *t*-tests.

## Results

### Behavioral Results

**Figure 2:**
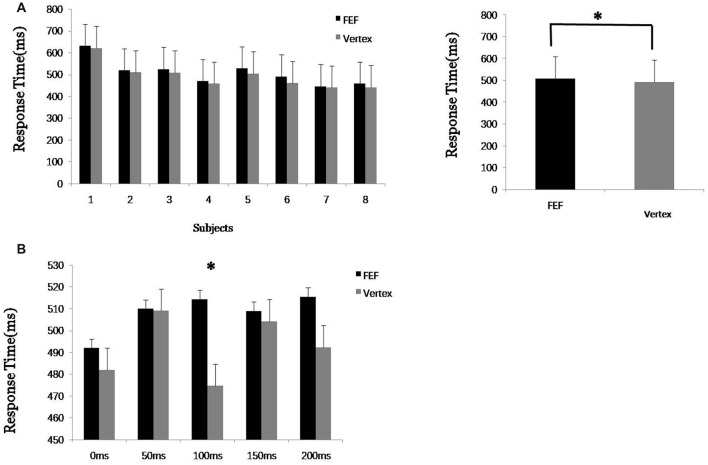
Experiment 1 results. **(A)** Average response time of each eight subjects to transcranial magnetic stimulation (TMS) applied to the right frontal eye field (FEF) and vertex. **(B)** Response time after TMS at 0, 50, 100, 150 and 200 ms from the beginning of the first visual target stimulus. “*” means significantly different (*p* < 0.05).

In the first experiment, data from eight subjects were analyzed (one subjects’ data were excluded because he could not focus on the visual FBA task for a sufficiently long time). First, the response times when TMS was applied to the rFEF and vertex were statistically analyzed. To mitigate the effect of TMS being applied at different times, we averaged the response times of five groups of trials (TMS at 0, 50, 100, 150 and 200 ms from the beginning of the first visual target stimulus). The results showed that when TMS was applied to the rFEF, the response time was slightly longer than when TMS was applied to the vertex, and this difference was significant (*p* < 0.05*, Figure 2). Thus, TMS applied to the FEF compared to TMS applied to the vertex led to interference in the behavioral response. This indicates that the FEF plays an important role in the process of visual FBA.

The reaction time when TMS was applied at different time points (0, 50, 100, 150 and 200 ms) was further analyzed. The result showed that the response times when TMS was applied to the FEF at different time points were all longer than when TMS was applied to the vertex, but the difference was only significant (*) for the 100 ms time point. This shows that TMS leads to inhibition and the action time of the FEF regarding the visual FBA process occurs mainly at around 100 ms. This result also provided an important time parameter for TMS use in the second experiment. Thus, in the second experiment, single-pulse TMS was applied at 100 ms after the target image stimulus began, which maximized the effect on the FEF, making it more convenient to study the spatial transmission of the FEF signal. One-hundred milliseconds also corresponds to the beginning of the second visual stimulus picture (out of six pictures).

The results in Table 1 show that, in the first experiment, when TMS was applied to the FEF at 100 ms, the response times (for both color and motion) were significantly changed (*p* = 0.003 and 0.009, respectively) compared to the response times in the control trials (which involved TMS applied to the vertex). The results show that TMS significantly inhibited the FEF and thus inhibited the assessment of the two attributes (color and motion). The variance analysis between the two groups (color/motion × FEF/vertex), shown in the final column of Table 1, shows that there was no significantly difference between the feature attributes and the TMS stimulus sites. Furthermore, we cannot find the difference between color and motion through the response time of TMS on FEF and on vertex.

**Table 1:** Variance analysis results for response time in feature attributes (color/motion) and TMS stimulus sites (FEF/vertex).

	FEF × vertex (color)	FEF × vertex (motion)	Feature × site
*p* value	0.003*	0.009*	0.916

“*” means significantly different (p < 0.05).

In the second experiment, TMS was applied at 100 ms after the target image stimulus began, and there was no significant difference in response time between the TMS and no TMS trials (*p* > 0.05). Thus, the effect of TMS only regard as an increase of the signal amplitude that could not change the neural function of the stimulus site (i.e., the FEF), to explore the spatial transmission of the FEF signal.

### TMS Combined With EEG Recordings

**Figure 3:**
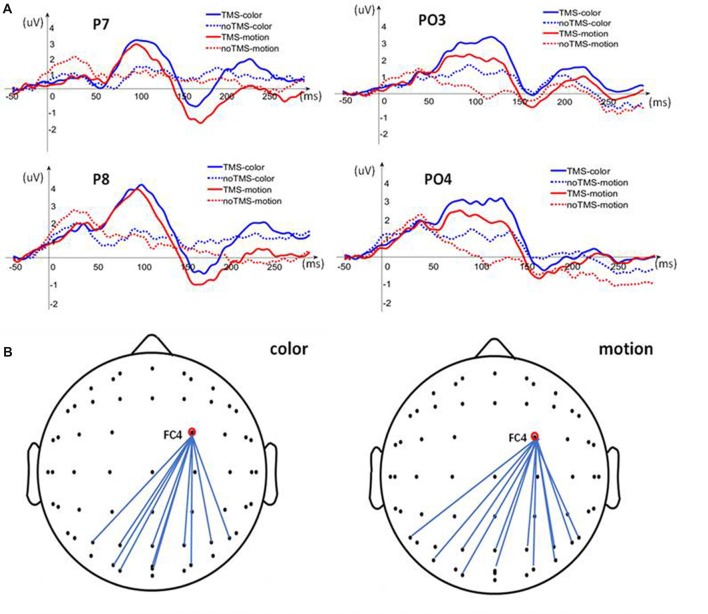
Analysis of signal amplitudes. **(A)** Posterior electrode (P7, P8, PO3 and PO4) signal amplitude compared between the TMS-event-related potential (ERP) and no TMS-ERP trials for both color and motion. **(B)** Pearson correlation analysis between FC4 (corresponding to the rFEF) and electrodes in the parietal and posterior brain regions.

**Figure 4:**
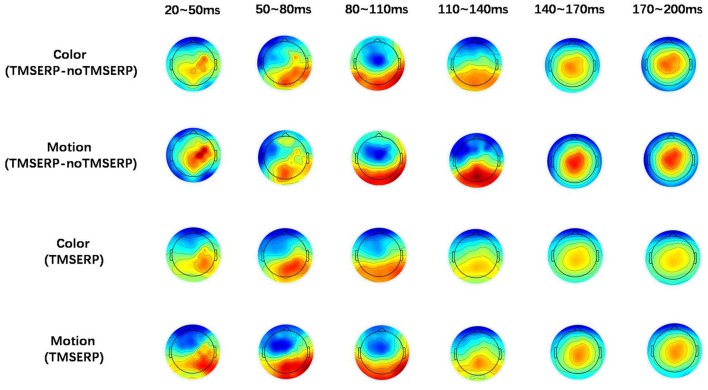
Brain topographic maps. The upper two rows show brain topographic maps of the difference between TMS-ERP and no TMS-ERP during color and motion FBA tasks, respectively. The lower two rows show the brain topographic maps associated with TMS-ERP.

In the second experiment, after TMS applied to the rFEF, it was found that the amplitude of FC4 (corresponding to the rFEF) increased immediately during color and motion attention. The electrode signal amplitude in the posterior brain region also increased, but the rise in the amplitude lagged by ~50 ms compared with that of FC4 (Figure 3A). To study the increase in the signal amplitude during the delayed time period, we further analyzed the Pearson correlation between the FC4 and the parietal and posterior brain regions at 20–50 ms after TMS, Figure 3B. We found that there was a strong correlation in the electrode signal amplitudes between many of the brain regions during both the color and motion FBA tasks. However, there were some differences regarding the electrodes for which there were significant Pearson correlations (color: P3, P4, 01, PZ, P1, P2, PO3, PO4, P6, POZ and OZ; motion: P4, O1, P7, P8, PZ, P1, P2, PO4, P6, PO7 and PO8 and POZ). These slight differences suggest that there are differences in neural signal transmission between color and motion FBA tasks, Figure 4.

To further explore the transmission direction of the signal from the rFEF after TMS, we divided the post-TMS EEG data (20–200 ms) into six periods containing 30 ms each. The effect of TMS was obtained by subtracting the no TMS-ERP recordings from the TMS-ERP recordings. The brain topographic maps associated with TMS, as shown in Figure 4, show that neural excitability was transferred from FC4 (near the stimulation point) to the posterior part of the brain at both 20–50 ms and 50–80 ms. At 80–140 ms, the neural excitability was transmitted to the posterior area, then back to the parietal lobe after 140 ms and it stayed in the parietal lobe until 200 ms. The brain topographic maps associated with TMS-ERP were also analyzed, and we found that the transfer effect was mainly associated with the initial time period (20–50 ms), which was consistent with the result regarding the TMS effect, but the neural excitability was transmitted more to the right hemisphere.

**Figure 5:**
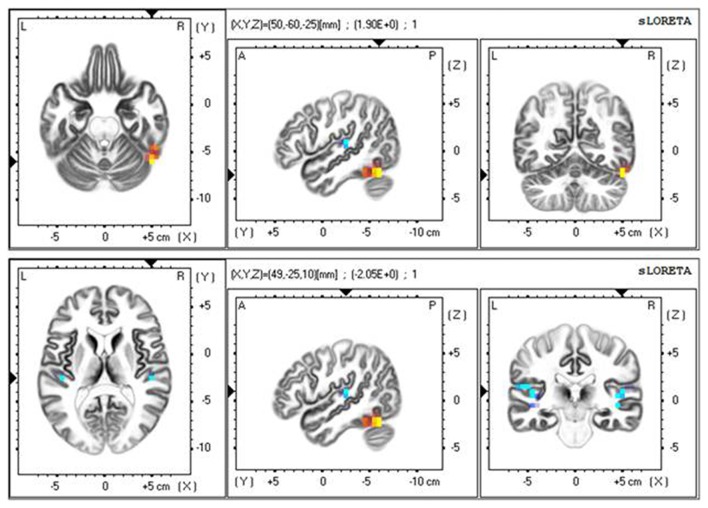
Standardized low-resolution brain electromagnetic tomography (sLORETA) results at 20–50 ms after TMS.

To further observe the spatial signal at 20–50 ms, we used a source location analysis method (sLORETA) to analyze the distribution of the current source density of the signal during the color and motion FBA tasks. We analyzed the difference between color and motion using paired *t*-tests in the statistical module of sLORETA. Current source density analyzed results at 20–50 ms after TMS are showed in Figure 5. The main positive distribution was in the fusiform gyrus (BA37), and the negative distribution was near the bilateral junction of the parietal and temporal lobes. The results show that the processing of different attributes in the posterior brain region is different, which concurs with previous researches showing that color processing is associated with V4 and motion processing is associated with MT/V5 (Zhou and Desimone, 2011; Alexander et al., 2018).

## Discussion

In this study, we analyzed the action time of the FEF during visual FBA and the spatial transmission of the FEF signal. In the first experiment, we applied single-pulse TMS above the threshold (110% RMT). The response time of the subject was interfered when TMS was applied to the FEF compared with when TMS was applied to the vertex. Thus, we verified the important effect of the FEF in the visual FBA process. Additionally, we explored different TMS stimulus times and found that the effective action time of the FEF occurred during the early stage of attention, which was about 100 ms after the target image stimulus began. These results not only provide information regarding the action time of the FEF, but they also provided an important time parameter for the study of the spatial transmission of FEF signals in the second experiment. The duration of the effect of single-pulse TMS is shorter than that of paired-pulse TMS (O’Shea et al., 2004), which made our study of the FEF action time more accurate. This method (i.e., the use of single-pulse TMS) is of great significance to the study of visual attention and even to other neurocognitive studies.

The FEF signal, which is also called the top-down signal, has been thought to come from the prefrontal lobe and to be transferred to the posterior brain region. In our study, we applied single-pulse TMS below the stimulus threshold (70% AMT) without changing the function of the stimulus site (i.e., the FEF), as we consider the neural activity transfer as the superposition of a nerve signal in the stimulus brain region. By assessing the superimposed signal, the process of neural signal transmission can be accessed directly and effectively; this method was applied and verified in a previous study (Johnson et al., 2012). This article provides evidence on FEF signal transmission during the assessment of different visual attributes. Single-pulse TMS combined with EEG recording has a high degree of time resolution, is quite effective for studying signal transmission processes, and is of great significance for research on the visual FBA mechanism.

Regarding the study of different attributes involved in visual FBA, there have been a large number of studies on color and motion. Different brain regions process color and motion information separately. V4, which is located near the fusiform gyrus, has mostly been shown to be responsible for processing color information. The V5/MT region, which is mainly located at the bilateral intersection of the parietal and temporal lobes, is mainly responsible for processing movement information (Lechak and Leber, 2012). In our study, we also found that there was a difference in the signal of the posterior brain area during the processing of different visual attributes. This result provides further support regarding the independance of color and motion information processing. At the same time, the difference of the signals in the posterior region was might closely related to the FEF during the process of visual FBA.

Our study not only analyzed the action time of the FEF from a behavioral perspective, but it also explored the spatial transmission of the FEF signal by combining single-pulse TMS with EEG recordings. The application of single-pulse TMS with different parameters in two experiments shows the various effects of TMS and indicates that TMS could be used to achieve different research objectives. The study also provides further evidence regarding the transmission of FEF signals. The study is of great importance for research on visual FBA, and it also provides details of an effective method for use in other neurocognitive studies.

## Author Contributions

XC, J-NJ and FX were responsible for the design of the work, and completed the two experiments. XC and J-NJ completed the analysis and the interpretation of the data. XC, Z-PL and TY were responsible for drafting the manuscript.

## Conflict of Interest Statement

The authors declare that the research was conducted in the absence of any commercial or financial relationships that could be construed as a potential conflict of interest.
